# Recent Developments on Modeling for a 3-DOF Micro-Hand Based on AI Methods

**DOI:** 10.3390/mi11090792

**Published:** 2020-08-21

**Authors:** Shuhei Kawamura, Mingcong Deng

**Affiliations:** Department of Electrical and Electronic Engineering, Graduate School of Engineering, Tokyo University of Agriculture and Technology, 2-24-16 Nakacho, Koganei-shi, Tokyo 184-8588, Japan; s195704q@st.go.tuat.ac.jp

**Keywords:** model, 3-DOF micro-hand, actuator, nonlinear, support vector machine, multi-output support vector regression, ant colony optimization

## Abstract

Recently, soft actuators have been expected to have many applications in various fields. Most of the actuators are composed of flexible materials and driven by air pressure. The 3-DOF micro-hand, which is a kind of soft actuator, can realize a three degrees of freedom motion by changing the applied air pressure pattern. However, the input–output relation is nonlinear and complicated. In previous research, a model of the micro-hand was proposed, but an error between the model and the experimental results was large. In this paper, modeling for the micro-hand is proposed by using multi-output support vector regression (MSVR) and ant colony optimization (ACO), which is one of the artificial intelligence (AI) methods. MSVR estimates the input–output relation of the micro-hand. ACO optimizes the parameters of the MSVR model.

## 1. Introduction

Many robots have been used in industrial fields; for example, they assemble a car, transfer wafer, and so on. In recent years, robots have been expected to be used not only for such fields but also for medical and welfare fields. In these fields, robots are required to handle human bodies and objects carefully.

To realize such robots, soft actuators have been getting increased attention. Soft actuators are made of flexible materials; for example, silicone rubber and synthetic resin; therefore, they can handle human bodies and objects delicately [1]. Various types of soft actuators have been developed and many of which are driven by air pressure. Many pneumatic soft actuators are composed of tubes that show expansion and bending motions under air pressure [2]. For example, there are McKibben pneumatic artificial muscle [3,4,5], a flexible micro actuator (FMA) [6], and a miniature pneumatic bending rubber actuator [7,8,9,10]. When air pressure is applied, the McKibben pneumatic artificial muscle expands in the radial direction and contracts in the longitudinal direction. However, the McKibben muscle can contract only in the longitudinal direction. Bending motions are difficult for the muscle [2].

To solve these problems, a 3-DOF micro-hand has been invented by S. Wakimoto [11]. The micro-hand is small, thin and flat; therefore, it is expected to be applied as a tip of forceps or an endoscope for medical fields, especially in operations. In that case, the micro-hand is assumed to be used inside a human body and a position of the micro-hand is difficult to measure directly. The reason is, there are no sensors that can attach itself to the micro-hand because the micro-hand is too small. The micro-hand is 50 mm in length, 5.0 mm in width and 2.6 mm in thickness. The micro-hand should be controlled without sensors; therefore, the input–output relation of the micro-hand should be modeled as accurately as possible. On the other hand, the micro-hand has a nonlinearity by its structures and materials. Thus, the relationship is complicated. In previous articles [12], a model of the micro-hand has been proposed; however, it needs to be improved because of the differences between the previous model and the experimental results. For these reasons, it is necessary to model the micro-hand more accurately for control without sensors.

In this paper, artificial intelligence (AI) is utilized for modeling the input–output relation of the micro-hand. It is difficult to model a control target with nonlinear characteristics, such as soft actuators, by using physical relationships, and there are often large errors between the actual measured values and the model. On the other hand, AI methods can model even a control target with a nonlinearity because the input–output data obtained from experiments are utilized. Furthermore, methods such as using physical relationships take a lot of time, because the relation is first modeled and then compared with the experimental data to check the accuracy of the model. In contrast, AI methods use only the experimental data to model, therefore it does not take much time. In addition, AI methods can be easily applied to other control objects because the modeling procedure does not change much. In recent years, AI has been applied to robotic actuators for quality and reliability improvement, reduction in cycle time, floor space utilization, and so on [13,14]. AI methods also have been used for estimating a model of soft actuators. A miniature pneumatic bending rubber actuator can be controlled without sensors by using support vector regression (SVR), which is one of the machine learning methods [7,10] and a regression machine that is extended from a support vector machine (SVM) [15,16]. SVR has a high generalization ability with few training data and is valid for a nonlinear model. In spite of its potential usefulness, the standard SVR cannot cope with multi-output problems. The input–output relation of a miniature pneumatic bending rubber actuator is a single-input single-output (SISO) system, therefore there is no problem with applying the standard SVR. In contrast, the relation of the micro-hand is a three-input three-output system, therefore it is necessary to use a different SVR for each of the three outputs. This approach, however, ignores cross relations between the outputs and makes it difficult to select parameters because the number of parameters is increased. To deal with this problem, a multi-output support vector regression (MSVR), which extends SVR to multiple outputs, is proposed [17]. In this article, MSVR is used for modeling the micro-hand. In addition, selecting optimal parameters is needed to improve the estimation ability of MSVR. In this paper, ant colony optimization (ACO) [18,19,20,21] is used for selecting the parameters. ACO was devised from the behavior of real ants as they move from their nest to a food source and has been utilized to solve complex combinatorial optimization problems [18]. Also, ACO has only two parameters; therefore, using ACO makes selecting the parameters of MSVR easier. The relation is modeled by using MSVR with ACO. Moreover, the proposed model is compared with the experimental results and the previous model.

## 2. Materials and Methods

This section describes methods for modeling the input–output relation of a 3-DOF micro-hand. In Section 2.1, the structure of the micro-hand is shown. In Section 2.2, the previous method for modeling the micro-hand is introduced. In Section 2.3, the proposed method for modeling the relation by using MSVR and ACO is presented. In Section 2.3.1, the standard SVR and MSVR are introduced. MSVR is utilized for modeling the relation. In Section 2.3.2, the ACO algorithm for selecting the parameters of MSVR is presented. In Section 2.3.3, an AI–based experimental system and flow are shown. The experimental system is used to obtain the input–output data of the micro-hand and model the relation.

### 2.1. The Structure of the 3-DOF Micro-Hand

**Figure 1:**
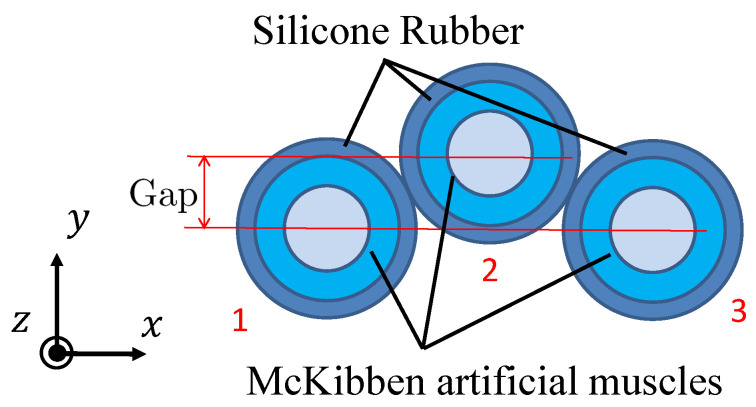
The cross section of the 3-DOF micro-hand.

**Figure 2:**
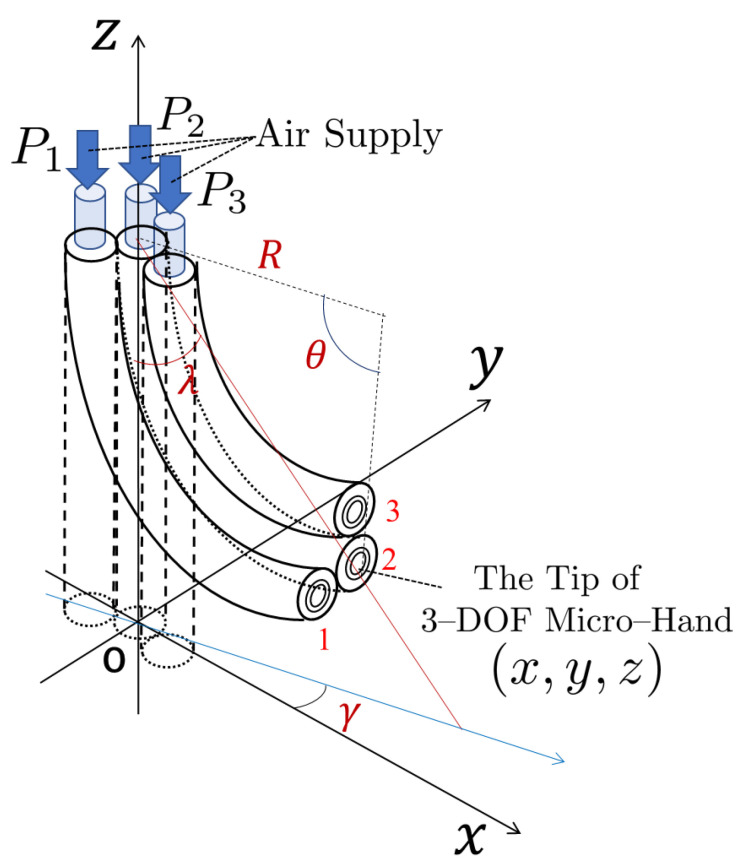
The coordinate system for 3-DOF micro-hand.

The cross section of the 3-DOF micro-hand is shown in Figure 1. The micro-hand has three McKibben artificial muscles arranged in parallel [11]. The muscle is shaped like a rubber tube, closed at one end, and contracts in the axial direction when air pressure is applied to the inside [2]. The *x*, *y*, and *z* axes and muscles 1, 2, and 3 are defined, as shown in Figure 1 and Figure 2. Silicone rubber covers and bonds the three muscles. There is a gap between muscle 2 and the other muscles 1 and 3. These structures allow the micro-hand to bend in any direction by changing the input air pressure pattern to the muscles [12]. $P_{1}$, $P_{2}$, and $P_{3}$ are the input pressures of the muscle 1, 2, and 3, respectively. In the experiment, the coordinates of the tip of the micro-hand (x,y,z) are measured when pneumatic pressure is applied. θ is a bending angle of the micro-hand, γ is a bending direction angle, *R* is a curvature radius of the central axis of the micro-hand, and λ is an angle between *z*-axis and a straight line passing through the tip and the bottom of the micro-hand. θ, γ, *R*, and λ are utilized for the previous method.

### 2.2. Previous Method

**Figure 3:**
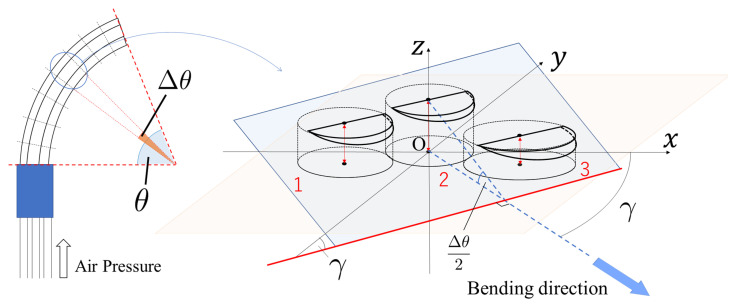
A piece of the divided micro-hand.

The previous model is derived by using physical and geometrical methods. The bending micro-hand is divided into the axial direction, as shown in Figure 3, for modeling the relation of the micro-hand. The piece consists of three cylinders which have different axial lengths and diameters. By considering the piece, θ, γ, and *R* are derived [12].

(1) $$\begin{array}{cc} \theta & =2n\sqrt{2\left(1-\frac{c}{\sqrt{a^{2}+b^{2}+c^{2}}}\right)}, \end{array}$$

(2) $$\begin{array}{cc} \;\gamma & =\left\{\begin{array}{c} \arccos\left(\frac{a}{\sqrt{a^{2}+b^{2}}}\right),\;0\leq b\\ \;\\ -\arccos\left(\frac{a}{\sqrt{a^{2}+b^{2}}}\right),\;b<0 \end{array}\right. \end{array}$$

(3) $$\begin{array}{cc} \;R & =\frac{L_{1}+L_{2}+L_{3}}{3\theta }, \end{array}$$

where *n* is the number of divided pieces; a,b, and *c* are physical parameters that change with the input pressure $P_{1}$, $P_{2}$, and $P_{3}$ of the muscles; $L_{1},L_{2},$ and $L_{3}$ are the axial lengths of the muscle 1, 2, and 3, respectively (see details in [12]). From Figure 2, the relation between the coordinates of the tip of the micro-hand (x,y,z) and *R*, γ and λ are obtained. *R*, γ, and λ are transformed to *x*, *y*, and *z* geometrically as follows.

(4) $$\begin{array}{cc} x & =2R\sin^{2}\lambda \cos\gamma , \end{array}$$

(5) $$\begin{array}{cc} y & =2R\sin^{2}\lambda \sin\gamma , \end{array}$$

(6) $$\begin{array}{cc} z & =L_{0}-2R\sin\lambda \cos\lambda , \end{array}$$

(7) $$\begin{array}{cc} \lambda & =\frac{\theta }{2}. \end{array}$$

The detailed information about the previous method is written in [12].

### 2.3. Proposed Method

#### 2.3.1. Multi-Output Support Vector Regression

The micro-hand has a nonlinearity, thus, the input–output relation of the micro-hand is complicated, therefore we used multi-output support vector regression (MSVR) [17] for modeling the relationship in this paper. MSVR extends the standard SVR to multiple outputs and retains the advantage of a sparse and compact solution by utilizing the ε-insensitive loss-function [22]. The detailed information about the standard SVR and MSVR is written in [17].

The regression model f(x) is shown as

(8) $$\begin{array}{c} f\left(x\right)=w^{T}\phi \left(x\right)+b, \end{array}$$

where x means the input vector, w shows the weight vector, *b* is the offset and ϕ is a nonlinear function that maps the input space into a higher dimensional feature space [15]. The optimization problem in the standard SVR is finding regressor w and *b* that minimizes

(9) $$\begin{array}{c} \frac{{∥w∥}^{2}}{2}+C\sum_{i=1}^{n}L_{v}\left(y_{i}-\left({\phi \left(x_{i}\right)}^{T}w+b\right)\right), \end{array}$$

where $L_{v}\left(\;\cdot \;\right)$ is known as the ε-insensitive loss-function, which is equal to $\left|y_{i}-\left(\phi^{T}\left(x_{i}\right)w+b\right)\right|-\varepsilon$ for $\left|y_{i}-\left(\phi^{T}\left(x_{i}\right)w+b\right)\right|\geq \varepsilon$ and equal to 0 for $\left|y_{i}-\left(\phi^{T}\left(x_{i}\right)w+b\right)\right|<\varepsilon$. *C* is a penalty parameter and ε is an error accuracy parameter. The standard SVR can be solved using only inner products between $\phi \left(\;\cdot \;\right)$; therefore, it is only necessary to specify a kernel function $K\left(x_{i},x\right)={\phi \left(x_{i}\right)}^{T}\phi \left(x_{j}\right)$. In this paper, the RBF kernel is used as a kernel function and is shown as follows.

(10) $$\begin{array}{c} K\left(x_{i},x\right)=\exp\left(-\frac{{∥x_{i}-x∥}^{2}}{2\sigma^{2}}\right), \end{array}$$

where σ is a hyper-parameter in the RBF kernel.

The standard SVR is not able to solve the case when the output is a vector $y\in R^{Q}$, therefore MSVR needs to be applied. The MSVR solves the optimization problem by finding a regressor $w^{j}$ and $b^{j}\left(j=1,\ldots ,Q\right)$ for every output. This problem can be led to the minimization of

(11) $$\begin{array}{c} L_{P}\left(W,b\right)=\frac{1}{2}\sum_{j=1}^{Q}{∥w^{j}∥}^{2}+C\sum_{i=1}^{n}L\left(u_{i}\right) \end{array}$$

with respect to the weight vector W and the offset b, where $u_{i}=∥e_{i}∥=\sqrt{e_{i}^{T}e_{i}}$, $e_{i}^{T}=y_{i}^{T}-{\phi \left(x_{i}\right)}^{T}W-b^{T}$, $W=\left[w^{1},\ldots ,w^{Q}\right]$, $b={\left[b^{1},\ldots ,b^{Q}\right]}^{T}$. The ε-insensitive loss function can be extended to multiple dimensions, but it is based on an $L_{1}$ norm. The function based on an $L_{1}$ norm needs to consider each dimension independently and the solution complexity is in proportion to the number of dimensions. To solve the problem, by using an $L_{2}$ norm, all dimensions can be considered in a unique restriction that yields a single support vector for all dimensions [23]. The loss function based on an $L_{2}$ norm is proposed as follows.

(12) $$\begin{array}{c} L\left(u\right)=\left\{\begin{array}{cc} 0, & u<\varepsilon \\ u^{2}-2u\varepsilon +\varepsilon^{2}, & u\geq \varepsilon \end{array}\right. \end{array}$$

The relation of the micro-hand is a three-input three-output system; therefore, in this paper, $x=(P_{1},P_{2},P_{3})$ and y=(x,y,z) are the input vector and the output vector of the MSVR, respectively.

#### 2.3.2. Ant Colony Optimization

MSVR has penalty parameter C, error accuracy parameter ε, and hyper-parameter σ in RBF kernel function [17]. The three parameters (C, ε, and σ) have a main influence on the regression model precision. C determines the balance between model complexity and model precision. ε controls the sparsity of the support vector. σ reflects the distribution features of the training data and confirms the width of the local neighborhood. Grid search is often used to select these parameters; however, grid search requires a lot of calculation and time. To solve this problem, different kinds of classical techniques have been developed [10]. Among them, the meta-heuristic based methods (such as the genetic algorithm, the differential evolution algorithm, and the particle swarm optimization algorithm) are some of the most popular methods used to optimize the parameters as a multidimensional optimization problem [24,25]. In this paper, the ant colony optimization (ACO) algorithm is utilized for selecting optimal parameters of MSVR. ACO has only two parameters, therefore using ACO makes selecting the hyper-parameters of MSVR easier.

The ACO algorithm is inspired by the behavior of real ants moving from their nests to a food source [18,26,27]. ACO has been applied to many combinatorial optimization problems such as the traveling salesman problems (TSP). It is known that real ants use pheromones to find the shortest routes from nests to food. Ants release the pheromone on the path they passed, and other ants are attracted to the pheromone and select the route according to the quantity of the pheromone. Furthermore, the pheromone tends to evaporate, therefore the pheromone remains only in the shortest path at last, and ants take the shortest routes [18]. The mathematical model of the ACO algorithm is described as follows.

At iteration *t*, the probability for ant *k* moving from node *i* to node *j* is

(13) $$\begin{array}{c} p_{ij}^{k}\left(t\right)=\frac{\left[\tau_{ij}\left(t\right)\right]\;\cdot \;{\left[\eta_{ij}\left(t\right)\right]}^{\beta }}{\sum_{s\in N_{i}^{k}}\left[\tau_{is}\left(t\right)\right]\;\cdot \;{\left[\eta_{is}\left(t\right)\right]}^{\beta }},\forall j\in N_{i}^{k}, \end{array}$$

where *s* is a set of nodes that ant *k* can choose; $N_{i}^{k}$ is a set of nodes that ant *k* at node *i* has never chosen; β is expected heuristic factor; $\tau_{ij}$ is the intensity of trail information on the path between node *i* to node *j*; $\eta_{ij}$ is heuristic information. In the case of TSP, $\eta_{ij}$ can be defined as the inverse number of the distance between node *i* and node *j*. The heuristic information is optional, and the setting of $\eta_{ij}=1$ means that no heuristic information is considered [18]. In this paper, let $\eta_{ij}=1$, because the ACO algorithm is utilized for selecting parameters of MSVR and there is no relation between the value of one digit of the parameters and the value of the next digit.

The intensity of trail information on the path between node *i* to node *j* is

(14) $$\begin{array}{c} \begin{array}{c} \tau_{ij}\left(t+1\right)=\left(1-\rho \right)\tau_{ij}\left(t\right)+\sum_{k=1}^{m}\Delta \tau_{ij}^{k}\left(t\right)\\ \Delta \tau_{ij}^{k}=\left\{\begin{array}{cc} \frac{1}{L_{k}}, & \;\mathrm{if}\;\left(i,j\right)\in T_{k}\\ 0, & \;\mathrm{otherwise}\; \end{array}\right. \end{array} \end{array}$$

where ρ(0<ρ<1) is a pheromone volatile coefficient; *m* is the total number of ants; $\Delta \tau_{ij}$ is the pheromone quantity left on the path (i,j) by ant *k*; $L_{k}$ is the length of the path $T_{k}$ which ant *k* has moved. $\Delta \tau_{ij}$ is the inverse of $L_{k}$ so that the shorter $L_{k}$ is, the greater the value is. In this paper, the total number of ants is 20. In the initial state, it is assumed that every path has the same intensity of the pheromone $\tau_{0}$, therefore the initial pheromone is $\tau_{ij}\left(0\right)=\tau_{0}$.

In this paper, each digit of the parameters is represented by 10 nodes [28,29,30]. Thus, each digit contains 10 positive integers from 0 to 9. In this algorithm, penalty parameter C is 4-bit number, with range (0.0<C≤999.9); error accuracy parameter ε is 4-bit number, with range (0.0000<ε≤0.9999); hyper-parameter σ in RBF kernel function is 4-bit number, with range (0.000<σ≤9.999). When the three parameters are arranged in order, the bit number is 12 bits in total. The bit number can be represented as the character string $d_{0}d_{1}d_{2}\ldots d_{l-2}d_{l-1}$, in which *l* is the precision. Then, the string is converted into numbers according to the range of each parameter as follows.

(15) $$\begin{array}{c} x=\sum_{i=1}^{l}d_{\left(i\right)}*10^{-i}, \end{array}$$

where *x* is the converted variable; $d_{\left(i\right)}$ is the *i*th character in the string.

**Figure 4:**
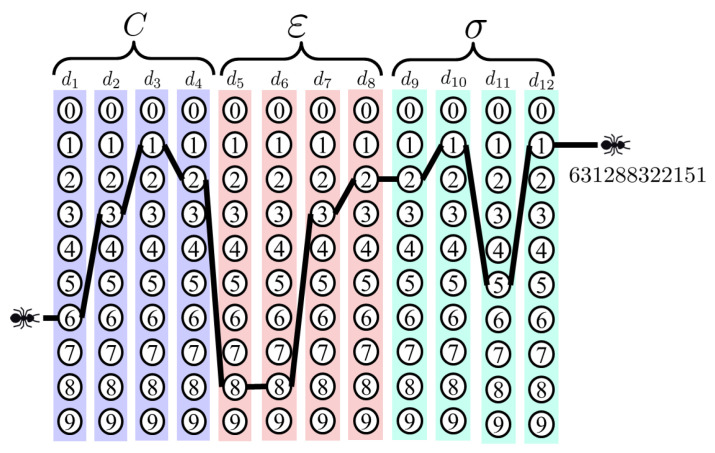
Parameters of the multi-output support vector regression (MSVR) model searched by an ant.

Figure 4 shows the above idea, in which every node can only be the value of 0–9 and the three parameters are arranged in order. As shown in Figure 4, the path connecting 12 nodes stands for the 12 digits of the character string, 631288322151, and the character string is converted into C=631.2,ε=0.8832, and σ=2.151 by using Equation (15).

Every optimization process is recorded in a one-dimension array. The following describes the steps to optimize MSVR parameters with ACO.

1. Set ant number *m*, the coefficient representing pheromone evaporation ρ, the initial pheromone $\tau_{0}$, time counter t=0, cycle number N=0, the maximum cycling times $N_{max}=80$, and a one-dimension array $A_{k}$.
2. Calculate the probability that the ant moves to each path node with Equation (13). Move the ant to the selected node, and record the coordinate value in the element *i* of $A_{k}$.
3. Set i=i+1, if ant *k* goes through 12 nodes, jump to (4), otherwise (2).
4. Set k=k+1, if all the ants go through 12 nodes, jump to (5), otherwise (2).
5. Obtain MSVR parameters by using $A_{k}$ and calculate mean absolute error (MAE) between experimental data and the MSVR model.
6. Update pheromone with Equation (14), clear $A_{k}$, and set N=N+1.
7. If $N<N_{max}$ and every ant does not take the same path, jump to (2); if $N<N_{max}$, but every ant takes the same path, then MSVR parameters are optimized.

#### 2.3.3. Experimental System

**Figure 5:**
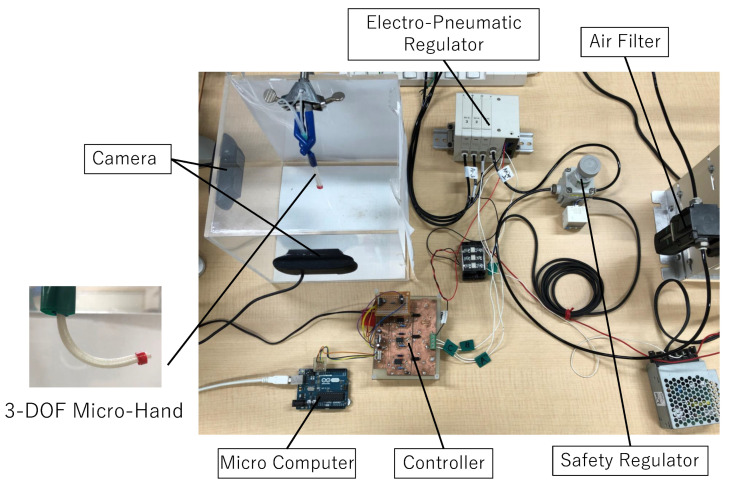
AI-based experimental system.

**Figure 6:**
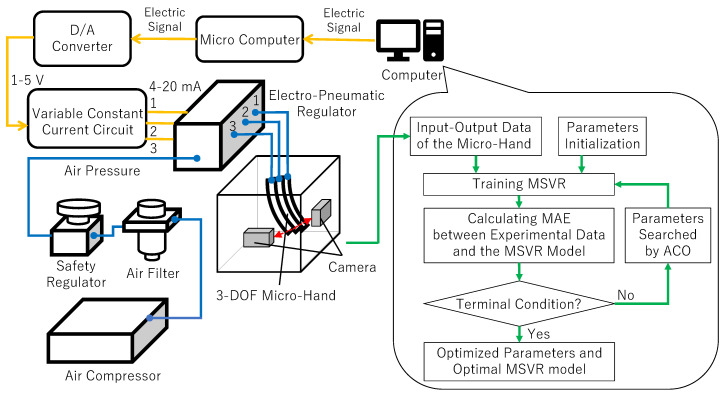
AI-based experimental flow.

In this section, an experimental system for obtaining the input–output data and modeling the input–output relation is introduced. Figure 5 shows the experimental system and Figure 6 shows the experimental flow [12]. The experimental system is configured by a 3-DOF micro-hand, an air compressor (0.20P-5S, HITACHI, Tokyo, Japan), an air filter (F1000-8, CKD, Aichi, Japan), a safety regulator (RP1000-8-07, CKD, Aichi, Japan), an electro-pneumatic regulator (MEVT500, CKD, Aichi, Japan), a controller for an electro-pneumatic regulator, two cameras (HD Pro Webcam C920r, Logicool, Tokyo, Japan), and a computer sending an electrical signal [12]. The following explains how to move the micro-hand and model the input–output relation of the micro-hand by the experiment.

1. The air compressor provides pneumatic pressure for the air filter and the filter sends clean air to the safety regulator.
2. The safety regulator limits the pressure to at most 300 kPa not to break the micro-hand.
3. The computer sends an electrical signal to the controller for controlling the electro-pneumatic regulator.
4. The controller provides 4–20 mA for the electro-pneumatic regulator and decides the aperture of the electro-pneumatic regulator.
5. Desired pressures are sent into the micro-hand and it bends or contracts.
6. The coordinates of the tip of the micro-hand are captured by two cameras.
7. The experimental data is sent to the computer and the input–output relation of the micro-hand is modeled by using MSVR and ACO.

## 3. Results and Discussion

This section shows and discusses the simulation results obtained by the proposed methods in Section 2. The simulation results are obtained using MATLAB(R2019a), which is one of the effective software products for system engineering. In Section 3.1, the parameters of MSVR are selected by ACO. In Section 3.2, the input–output relation of the micro-hand is modeled by MSVR.

### 3.1. The Parameters of Multi-Output Support Vector Regression (MSVR) Selected by Ant Colony Optimization (ACO)

ACO has two parameters that affect its performance in terms of solution quality. Table 1 shows parameters of ACO for selecting the optimal parameters of MSVR.

**Figure 7:**
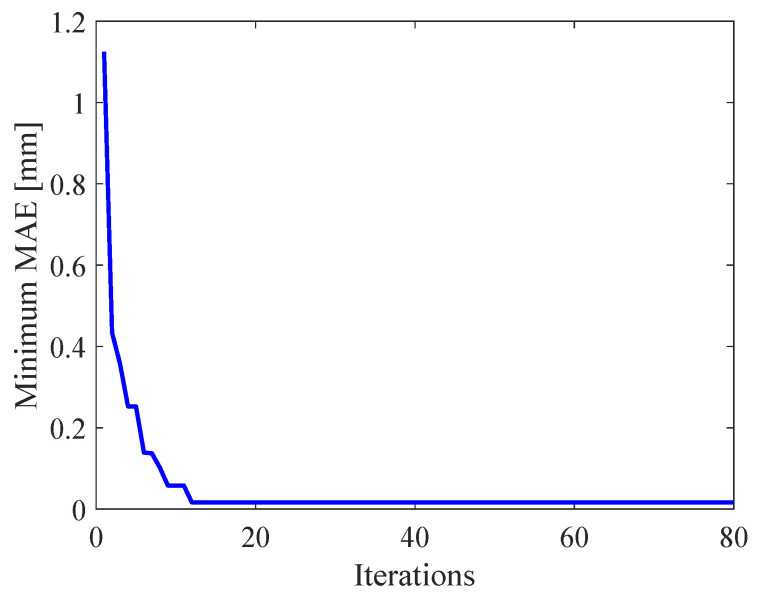
Relation between iterations and minimum mean absolute error (MAE).

**Figure 8:**
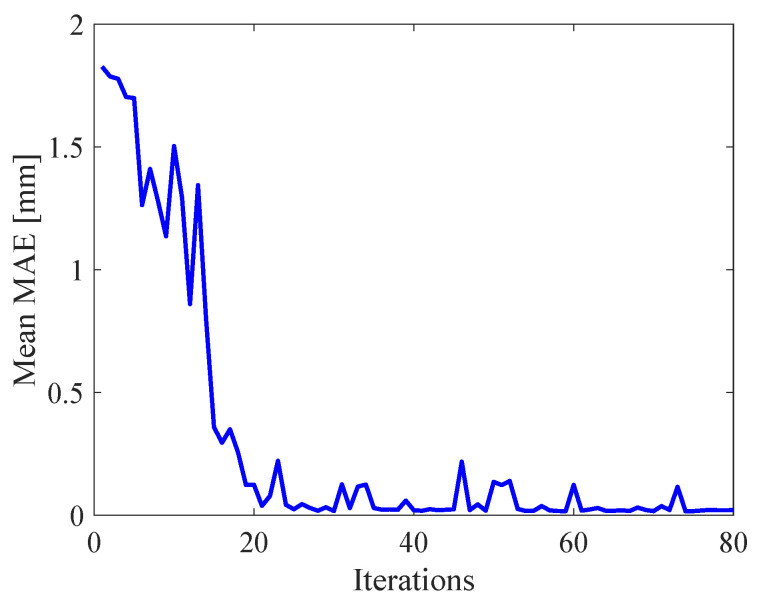
Relation between iterations and mean MAE.

The parameters of MSVR are evaluated by mean absolute error (MAE) between experimental results and the MSVR model. The process of obtaining optimal parameters of MSVR are shown in Figure 7 and Figure 8. Figure 7 shows the relation between iterations and the minimum MAE, and Figure 8 shows the relation between iterations and the mean MAE. In Figure 7, the MAE decreases and converges with increasing iterations. In Figure 8, the mean MAE decreases and converges with increasing iterations. The parameters of MSVR selected by ACO are shown in Table 2.

### 3.2. Model for 3-DOF Micro-Hand Estimated by MSVR

**Figure 9:**
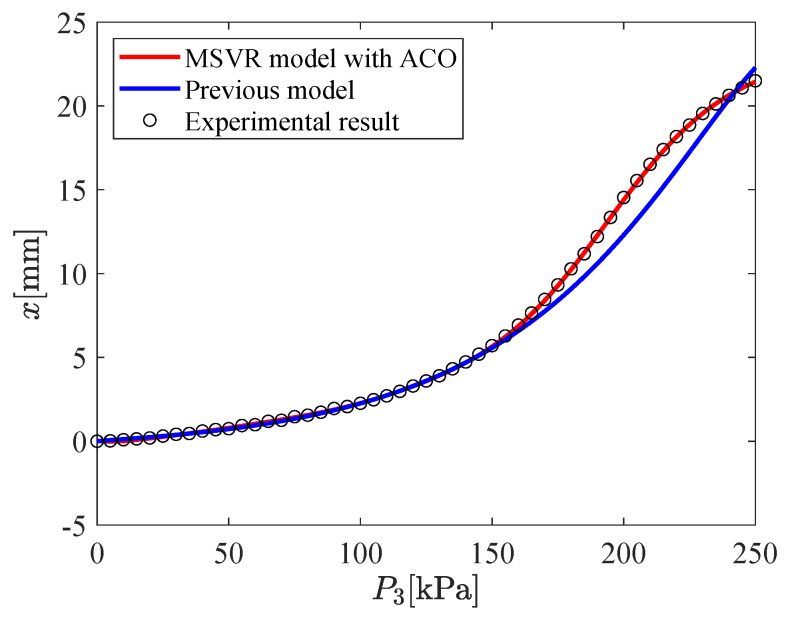
Relation between $P_{3}$ and *x* ($P_{1}=P_{2}=0\;\mathrm{kPa},P_{3}=$ 0 kPa–250 kPa).

**Figure 10:**
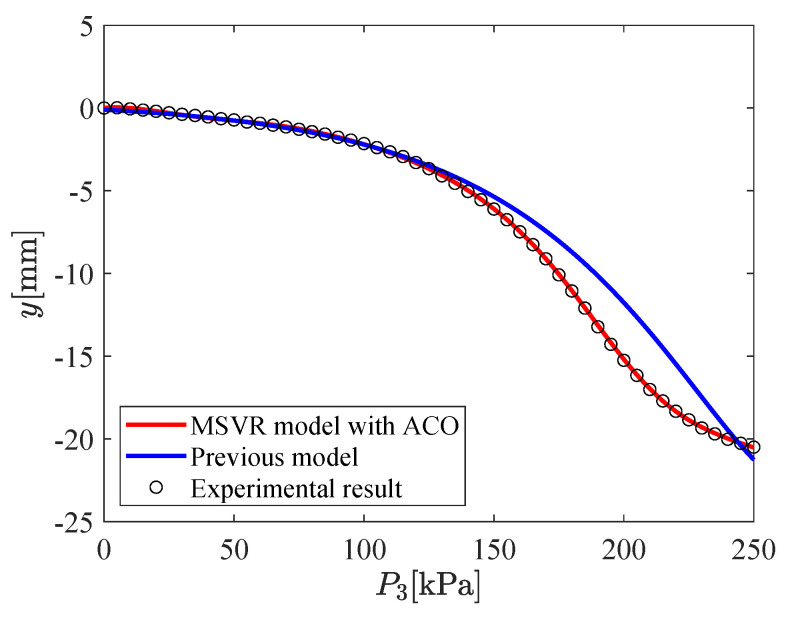
Relation between $P_{3}$ and *y* ($P_{1}=P_{2}=0\;\mathrm{kPa},P_{3}=$ 0 kPa–250 kPa).

**Figure 11:**
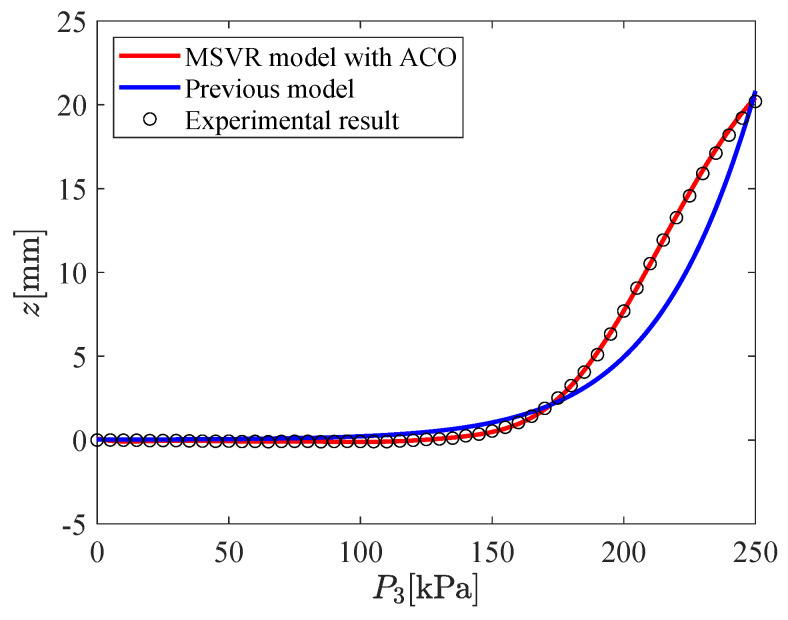
Relation between $P_{3}$ and *z* ($P_{1}=P_{2}=0\;\mathrm{kPa},P_{3}=$ 0 kPa–250 kPa).

**Figure 12:**
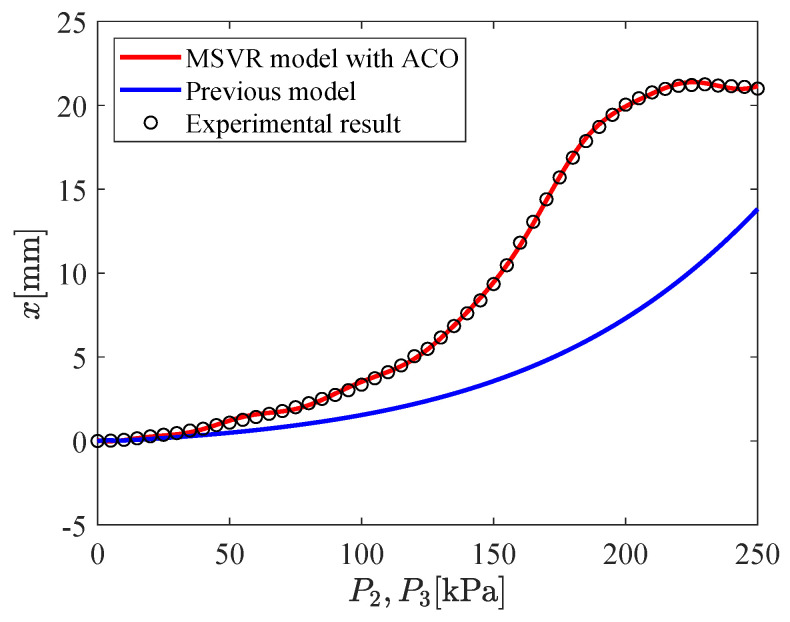
Relation between $P_{2},P_{3}$ and *x* ($P_{1}=0\;\mathrm{kPa},P_{2}=P_{3}=$ 0 kPa–250 kPa).

**Figure 13:**
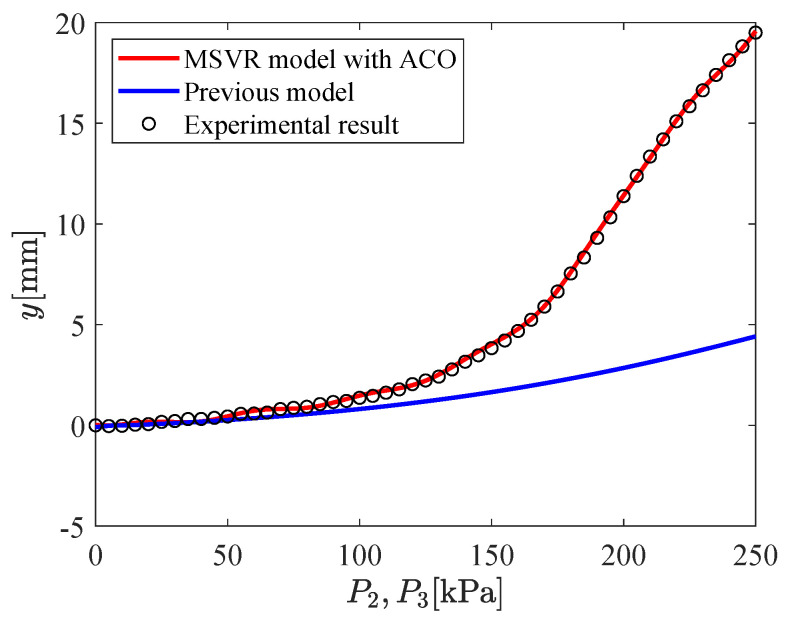
Relation between $P_{2},P_{3}$ and *y* ($P_{1}=0\;\mathrm{kPa},P_{2}=P_{3}=$ 0 kPa–250 kPa).

**Figure 14:**
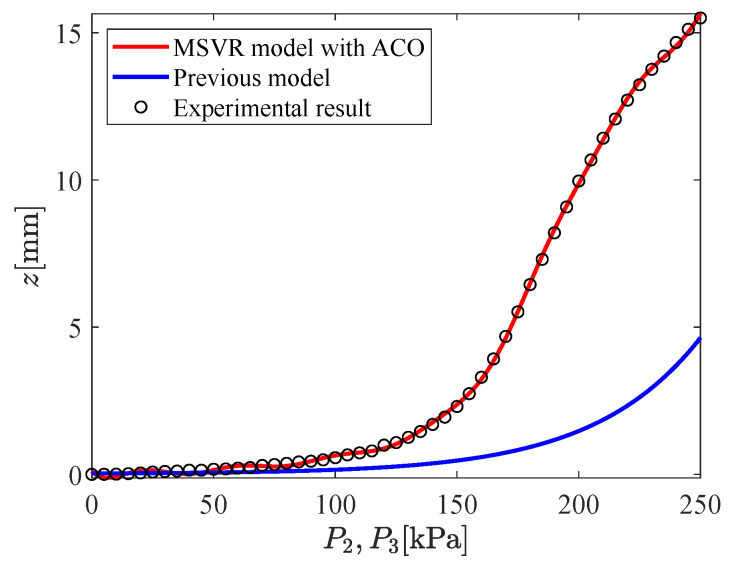
Relation between $P_{2},P_{3}$ and *z* ($P_{1}=0\;\mathrm{kPa},P_{2}=P_{3}=$ 0 kPa–250 kPa).

The input–output relation of the micro-hand is modeled by using MSVR with the parameters shown in Table 2. Figure 9, Figure 10, Figure 11, Figure 12, Figure 13 and Figure 14 show the proposed model (MSVR model with ACO), the previous model, and the experimental results. Experimental results are obtained by using two cameras. In Figure 9, Figure 10 and Figure 11, $P_{1}\;=\;P_{2}=0\;\mathrm{kPa}$ and $P_{3}=$ 0 kPa–250 kPa. In Figure 12, Figure 13 and Figure 14, $P_{1}=0\;\mathrm{kPa}$ and $P_{2}=P_{3}=$ 0 kPa–250 kPa. The previous model uses the same parameters and conditions as written in the previous article [12].

For comparing the proposed model and the previous model with experimental results, MAE is calculated. From Figure 9, Figure 10 and Figure 11, MAE of the proposed model is 0.0161 mm, while MAE of the previous model is 2.7048 mm. From Figure 12, Figure 13 and Figure 14, MAE of the proposed model is 0.0228 mm, while MAE of the previous model is 1.0506 mm. MAE of the proposed model is less than that of the previous model, therefore, the effectiveness of the proposed model is shown. Furthermore, it is considered that the proposed methods can estimate the inversion of the input–output relation. The inverse model is useful in designing a feedforward controller.

## 4. Conclusions

In this paper, AI methods for modeling the input–output relation of a 3-DOF micro-hand is proposed. MSVR is utilized for estimating the relation because it is a three-input three-output system and MSVR can deal with multiple outputs. In addition, optimal parameters of MSVR are selected by using ACO. The proposed model is compared with the previous model by the simulation, and the proposed model is more accurate than that of the previous model. In conclusion, the effectiveness of the proposed methods is shown. Since the proposed methods can be applied to any system with multiple-input multiple-output(MIMO) and nonlinear characteristics, it can be applied to any robotic system other than the micro-hand, as long as the input–output data are available. It helps to control objects that have not been used in the past due to the difficulty of modeling. Further studies are needed to design a control system without sensors for the micro-hand including the proposed model. Furthermore, it is considered that the control system cannot work properly without sensory feedback due to uncertainties and disturbances. To solve the problem, further studies are also needed to design a feedback control system with a sensor; for example, by attaching a micro-camera to the base of the micro-hand and obtaining the coordinates of the tip of the micro-hand, a robust control system could be designed by using image processing and the proposed method.

## Figures and Tables

**Table 1:** Parameters of ant colony optimization (ACO).

Parameter	Definition	Value
ρ	Evaporation coefficient	0.04
$\tau_{0}$	Quantity of initial pheromone	0.5

**Table 2:** Parameters of the MSVR model selected by ACO.

Parameter	Definition	Value
C	Penalty parameter	326.7
ε	Error accuracy parameter	0.0169
σ	Hyper-parameter in RBF kernel function	0.259

## Abbreviations

The following abbreviations are used in this manuscript:

DOF	Degrees of freedom
AI	Artificial Intelligence
FMA	Flexible micro actuator
SISO	Single-input single-output
MIMO	Multiple-input multiple-output
SVM	Support vector machine
SVR	Support vector regression
MSVR	Multi-output support vector regression
ACO	Ant colony optimization
TSP	Traveling salesman problems
RBF	Radial basis function
MAE	Mean absolute error
